# Potential Application of Peppermint (*Mentha piperita* L.), German Chamomile (*Matricaria chamomilla* L.) and Yarrow (*Achillea millefolium* L.) as Active Fillers in Natural Rubber Biocomposites

**DOI:** 10.3390/ijms22147530

**Published:** 2021-07-14

**Authors:** Marcin Masłowski, Andrii Aleksieiev, Justyna Miedzianowska, Krzysztof Strzelec

**Affiliations:** Institute of Polymer & Dye Technology, Lodz University of Technology, Stefanowskiego 12/16, 90-924 Lodz, Poland; andrii.aleksieiev@dokt.p.lodz.pl (A.A.); justyna.miedzianowska@edu.p.lodz.pl (J.M.); krzysztof.strzelec@p.lodz.pl (K.S.)

**Keywords:** natural rubber, peppermint, German chamomile, yarrow, biofillers, biocomposites, functional properties

## Abstract

In this study, peppermint (*Mentha piperita* L.), German chamomile (*Matricaria chamomilla* L.) and yarrow (*Achillea millefolium* L.) were applied as natural fibrous fillers to create biocomposites containing substances of plant origin. The purpose of the work was to investigate the activity and effectiveness of selected plants as a material for the modification of natural rubber composites. This research was the first approach to examine the usefulness of peppermint, German chamomile and yarrow in the field of polymer technology. Dried and ground plant particles were subjected to Fourier transmission infrared spectroscopy (FTIR) and UV–Vis spectroscopy, thermogravimetric analysis (TGA), goniometric measurements (contact angle) and scanning electron microscopy (SEM). The characterization of natural rubber composites filled with bio-additives was performed including rheometric measurements, FTIR, TGA, cross-linking density, mechanical properties and colour change after simulated aging processes. Composites filled with natural fillers showed improved barrier properties and mechanical strength. Moreover, an increase in the cross-linking density of the materials before and after the simulated aging processes, compared to the reference sample, was observed.

## 1. Introduction

Broad application of biocomposites in polymer materials processing and production was noticed over the last decades [1,2,3,4]. This trend occurs due to the multiple benefits related to the biocomposites production methods as well as their exploitation potential and ease of disposal [4,5]. Generally, it is noticeable that the main component of biocomposites (matrix) are biopolymers, thus replacing petroleum-derived materials [6,7,8]. Biocompatibility also applies to the selected reinforcement, which often comes from renewable sources. Hence, there is a noticeable increase in the importance of composite materials reinforced with natural fibers [9,10,11,12,13]. 

Natural rubber is a frequently used matrix for biocomposites since it is the only elastomer of natural origin with high availability and processing possibilities. It is used in polymer technology for the production of automotive parts, coated fabrics, belts, cables, rubber products, etc. [14]. Recently, many researchers have conducted studies to improve the properties of natural rubber composites in order to reduce the consumption of synthetic rubbers and polymers [15,16,17,18,19]. In many cases, the improvement of this type of biocomposite was achieved by adding physically and/or chemically modified natural fibrous fillers to the rubber mixture. Further research aims to find and characterize a type of natural filler that is cheap, widely available and has a strengthening effect on natural rubber-based biocomposite.

The most popular biocomposites with the addition of natural fiber are composites filled with wood, wood flour and other lignocellulosic fillers such as hemp, jute, bamboo, straw, horsetail, etc. [20,21,22,23]. These types of materials are favorable compared to the products with synthetic fibers due to their eco-friendly nature, low energy expenditure, low health risk and low emission of toxic vapors during processing, cheaper production and processing flexibility [24]. On the other hand, several drawbacks occur in fiber-reinforced composites production including low processing temperature, high moisture absorption and decreased durability of a final product [25]. In order to overcome them, various chemical and physical modifications of fibers were conducted to increase filler–matrix interactions and to obtain materials with improved processing and performance properties [16,26,27,28,29,30]. 

Due to the fact that previous studies of the authors proved the strengthening effect of lignocellulosic materials on the properties of biocomposites [31,32], a new challenge was undertaken—obtaining elastomer composites filled with herbal plants. The authors of the manuscript focused on three different herbs of the Asteraceae family as a biofiller for natural rubber biocomposites.

Peppermint (*Mentha piperita* L.) is a natural hybrid of *Mentha aquatica* L. and *Mentha spicata* L. [33]. The plant is cultivated worldwide but it also grows wild and spreads quickly in moist habitats, primarily in Europe, North America and Asia [34,35]. The largest producers and exporters of peppermint on the global market are the U.S.A., India, Japan and Great Britain [36]. Due to its wide occurrence all around the world, many different countries are also involved in the production of peppermint raw material and essential oil, including Germany, Russia, Italy, Bulgaria, Norway, Slovakia and Poland. *Mentha piperita* L. is known for its characteristic strong mint odor provided by menthol [37,38]. Previous studies of peppermint essential oils revealed their composition and therapeutic properties [39,40,41]. Besides menthol, it consists of menthone, menthyl acetate, carvone, linalool, limonene, pinene and others [42,43]. *Mentha piperita* L. is an antioxidant, antimicrobial, anti-allergic, antispasmodic, anticarcinogenic and antiviral herb [44,45,46,47,48,49]. These properties have ensured its wide application in various industries, including medicine, cosmetics and the food industry [50,51,52,53]. 

German chamomile (*Matricaria chamomilla* L., synonym: *Chamomilla recutita* L.) is an annual therapeutical herb cultivated in Europe, North Africa, Asia, North and South America, Australia, and New Zealand [54]. Previous studies proved its antioxidant, anti-cancer, neuroprotective, anti-inflammatory, antibacterial, anti-allergic, anti-diarrheal and antimicrobial effects [55,56,57,58,59]. *Matricaria chamomilla* L. is an official drug in pharmacopeia in 26 countries [54]. It is used as a treatment in various diseases and ailments such as skin irritations, bruises, rheumatic pain, rashes, chickenpox, ear and eye infections and nasal inflammation [60,61]. Moreover, it has applications in herbal teas, cough syrups and cosmetics (creams, ointments) [62,63,64,65]. The main constituents of the chamomile chemical composition are: α-bisabolol oxide, camphene, α-pinene, 1,8-cineole, camphor, chamazulene and flavonoids [66,67,68,69]. 

Yarrow (*Achillea millefolium* L.) is one of the oldest medicinal herbs growing wild and as a cultivated plant in the region of Eurasia and North America [70]. According to conducted studies, *Achillea millefolium* L. is a biologically active plant, which demonstrates multiple beneficial effects including antioxidant, anti-inflammatory, antispasmodic, hepatoprotective, antipyretic and antimicrobial activity [71,72,73,74]. The chemical composition of yarrow depends on the region of occurrence; however, it primarily consists of sabinene, 1,8-cineole, borneol, bornyl acetate, pinene and chamazulene [75,76,77,78]. *Achillea millefolium* L. helps eliminate toxins from the body, controls bleeding, lowers blood pressure, relieves menstrual pain and is used in the treatment of various diseases [79,80,81]. Moreover, it is used as a mouthwash to promote the healing of cuts [82] and a component of tea mixtures [83].

The purpose of this research article was to obtain natural rubber biocomposites filled with dried and ground peppermint, chamomile and yarrow to present their functionality as a lignocellulosic bio-filler in the elastomer technology. To provide a wide range of composites properties and herbs behaviour in obtained materials, several studies were conducted, including FTIR and UV–Vis spectroscopy, thermogravimetric analysis and contact angle measurement. Moreover, biocomposites were subjected to simulated thermo-oxidative and ultraviolet aging processes to examine mechanical properties, cross-linking density and colour change of non-aged and aged samples. Moreover, the rheological properties of elastomer mixtures were established. To determine the morphology of fillers and composites, scanning electron microscopy was used. The research presented in the manuscript is the first approach to use *Mentha piperita* L., *Matricaria chamomilla* L. and *Achillea millefolium* L. as a biofiller for natural rubber technology.

## 2. Results and Discussion

### 2.1. Characterization of Fillers

#### 2.1.1. Fourier Transform Infrared Spectroscopy (FTIR) 

**Figure 1:**
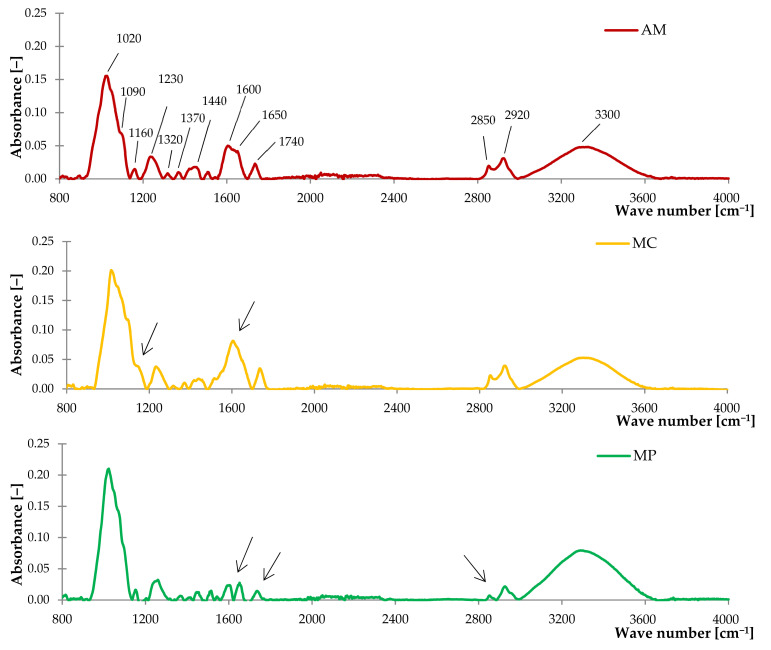
The FTIR spectra of yarrow (AM), German chamomile (MC) and peppermint (MP).

The obtained FTIR spectra were analyzed by assigning a wavelength value of registered peaks to corresponding functional groups (bonds) and by defining the type of vibration. The results are listed in Table 1 and FTIR spectra are shown in Figure 1.

The analysis of the FTIR spectra showed that examined fillers are characterized by the presence of similar functional groups with different peak intensities. A broad band registered in the range of 3600 to 3000 cm^−1^ corresponds to –OH groups of phenols and alcohols. Two peaks at 2850 cm^−1^ and 2920 cm^−1^ were assigned to stretching vibrations of symmetric and asymmetric aliphatic groups. Low-intensity peaks in the range of 1570 to 1230 cm^−1^ were assigned for amines, alkenes, aryl and methyl groups. On the other hand, an intensive peak at 1020 cm^−1^ was related to the C–O and C–C groups of cellulose [85]. Moreover, bands typical for lignocellulosic and hemicellulosic structures occur as several different peaks in the range of 1610 to 1150 cm^−1^ [86]. The groups of esters and aliphatic aldehydes were recorded based on the characteristic C=O vibration in the absorption range of 1730 to 1630 cm^−1^. At the FTIR spectrum of *Matricaria chamomilla* L., a maximum intensity was observed at 1600 to 1610 cm^−1^ corresponding to the C=C stretching vibrations of α,β-unsaturated ketones, which indicates the high content of these compounds in chamomile compared to *Mentha piperita* L., and *Achillea millefolium* L. The 1650 cm^−1^ peak was related to the vibration of the (–C=C–) and (–OH) groups, while the peak at 1740 cm^−1^ is typical for C=O vibrations of terpenes and terpenoids such as camphor, limonene, carvone, pinene [87]. Hence, the obtained spectra coincide with the FTIR studies carried out on various types of essential oils, which confirmed their content in the tested fillers [88,89].

#### 2.1.2. UV–Vis Spectroscopy

**Figure 2:**
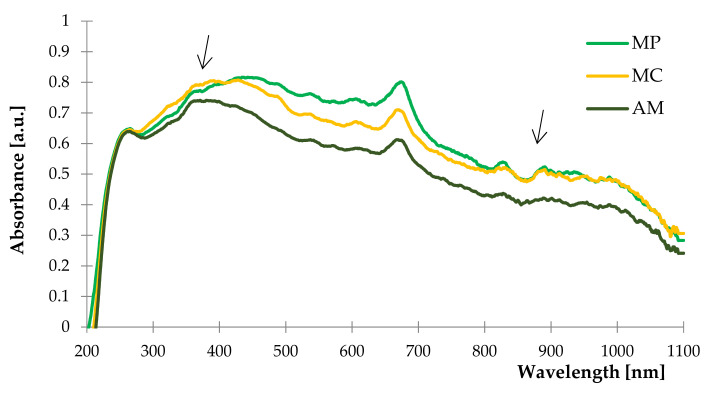
The UV–Vis spectra of peppermint (MP), German chamomile (MC) and yarrow (AM).

The results of the conducted study are presented in Figure 2 as UV–Vis spectra. The analysis revealed the content of similar compounds in each tested plant as it was observed that spectra curve over the same wavenumber range.

According to the previous research by Beigi et al. [41,67,75], peppermint, yarrow and chamomile are herbs with high contents of terpenes, terpenoids and phenolic compounds, which was confirmed by the peak registered at the 270 nm and as a band for 290–320 nm [90,91,92]. The chlorophyll content characteristic for plants was noticed as an extensive peak in the range of 360 to 450 nm and at 680 nm [93]. The absorption band recorded in the range of 400 to 500 nm was typical for the presence of carotenoids [94,95]. The results of UV–Vis spectroscopy were correlated to the FTIR spectra based on the identified classes of compounds.

The most significant difference in obtained spectra is the absorbance of the materials. In the case of *Matricaria chamomilla* L., the highest absorbance was recorded at 265 to 400 nm and in the range of 800 to 1100 nm, where both spectra of chamomile and peppermint overlapped. UV–Vis spectrum of yarrow characterized by the lowest absorbance over the entire wavelength range.

#### 2.1.3. Thermogravimetric Analysis

**Figure 3:**
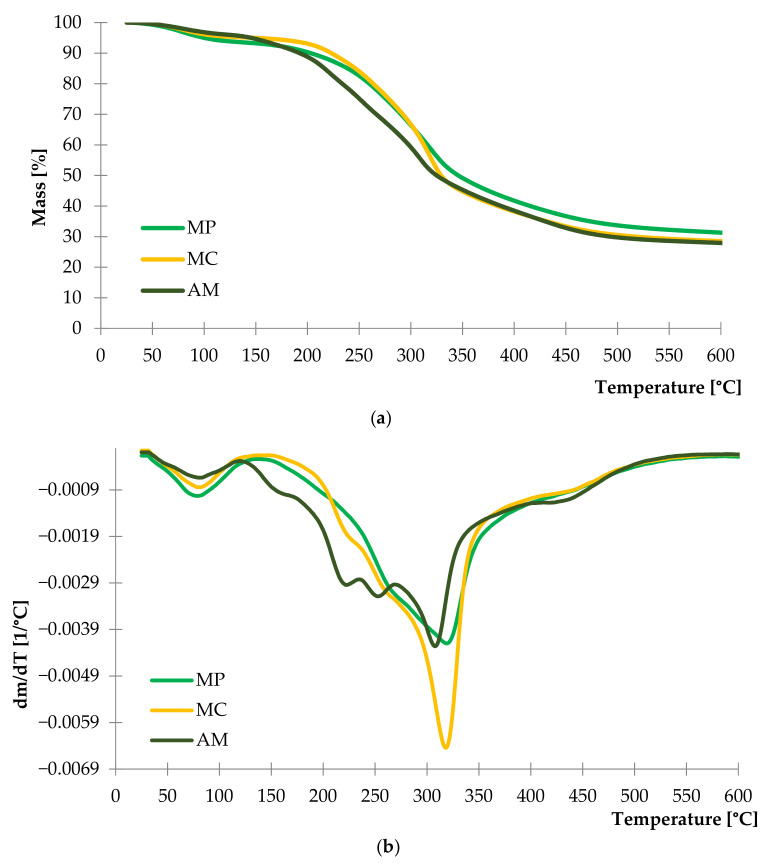
TG (**a**) and DTG (**b**) curves of peppermint (MP), German chamomile (MC) and yarrow (AM).

The thermal stability of herbal fillers was evaluated by thermogravimetric analysis. Results of the study are presented as TG and DTG curves in Figure 3. Characteristic parameters of conducted analysis are presented in Table 2.

The analysis of registered DTG curves showed that the first stage of thermal decomposition occurred in the range of 50 to 100 °C for each plant and it was related to the evaporation of water and moisture from fibers. Differences in the intensity of this mass loss for each sample indicated various content of water in plant structures. As a result, it can be stated that the peppermint has the highest water content in contrast to the yarrow. This was proved by the Δm_100_ parameter which was 4.99% and 3.18%, respectively for MP and AM samples (Table 2). The greatest mass loss was observed in the range of 165 to 425 °C with a maximum ca. 320 °C for all natural fillers. 

The thermal decomposition of yarrow began at ca. 165 °C. It was a relatively low temperature compared to the peppermint and chamomile, which began to decompose at 195 °C. According to these results, yarrow was a less thermally stable plant among other studied fillers. It may be related to the content of lignocellulosic materials in the herb’s structure as the thermal degradation of lignin and hemicellulose began at 160 °C, while in the case of cellulose it occurred at 220 °C [96]. Therefore, a multi-stage thermal process was recorded corresponding to the decomposition of each lignocellulosic material in samples. 

The results of parameter T_50_ and residue at 600 °C confirmed low thermal stability of the yarrow herbs in contrast to the peppermint. The DTG curve of chamomile presented the most intensive mass loss in the temperature range of 280 to 330 °C compared to the rest of the materials.

#### 2.1.4. Wetting and Contact Angle 

**Figure 4:**
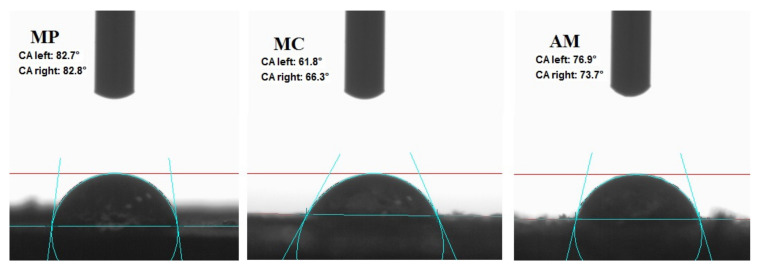
Contact angle (CA) measurements of peppermint (MP), German chamomile (MC) and yarrow (AM).

The hydrophilicity of *Achillea millefolium* L., *Matricaria chamomilla* L. and *Mentha piperita* L. was determined by measurement of the contact angle between the tablet and drop of water on its surface. The results are presented in the form of images in Figure 4. Wetting measurement provided information about the possible filler–matrix interactions and enabled to define the distribution of filler particles within polymer composite.

The presence of hydroxyl groups on the surface of the natural fillers indicated their polar and hydrophilic nature. This was confirmed by the obtained results as the contact angle of studied plants was below 90°. Again, these low values of contact angle were caused by the presence of terpenes and terpenoids in the herb composition such as menthol in *Mentha piperita* L. [36], bisabolol in *Matricaria chamomilla* L. [69] and cadinol or terpinen-4-ol in *Achillea millefolium* L. [75]. The lowest CA. 63° obtained for the sample of chamomile indicated the highest content of essential oil compounds and proved its increased hydrophilicity. This relatively low value of contact angle may decrease the distribution of filler in the non-polar polymer matrix and may cause weak interactions between hydrophilic plants and hydrophobic natural rubber.

#### 2.1.5. The Morphology of Fillers 

**Figure 5:**
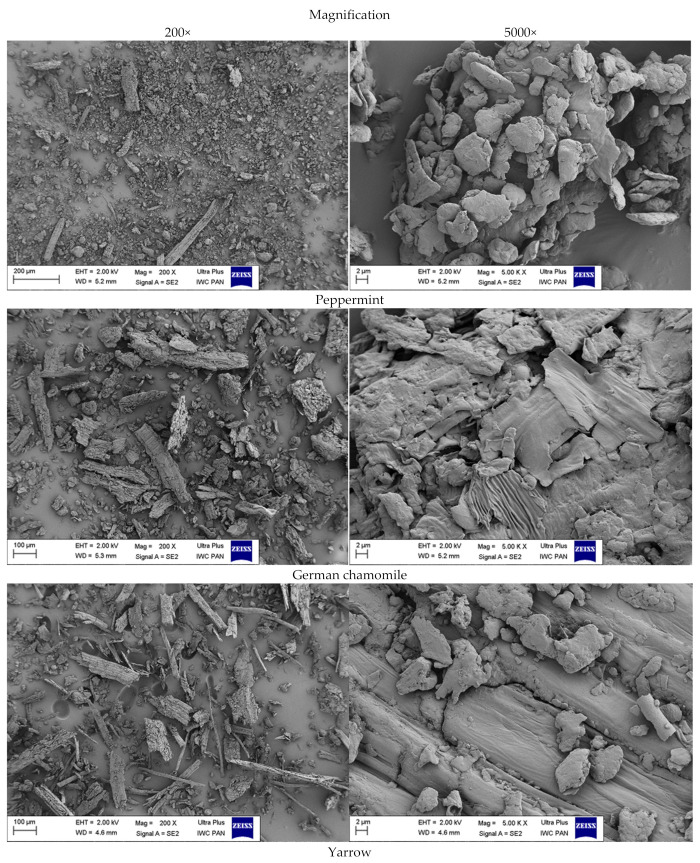
SEM images of fillers at the 200× and 5000× magnification.

Scanning electron microscopy (SEM) was used to determine the morphology, size and shape of dried and ground fillers. Images of peppermint, German chamomile and yarrow are presented in Figure 5 in the 200× and 5000× magnification.

The analysis of the structure of the bio-additives showed that materials after the grinding process varied in shape and size. Images at the 5000 magnification exposed the heterogeneous surface of tested materials. Moreover, the highest degree of fragmentation was obtained for peppermint. *Mentha piperita* L. consisted mainly of spherical small particles in the range of 5 to 20 µm with individual parts of greater dimension (100 to 300 µm) in the form of longitudinal fibers. Larger particles of thin and flat petals were obtained in both chamomile and yarrow. Among all tested plants, *Achillea millefolium* L. showed the most fibrous structure.

### 2.2. Characterization of Composites

#### 2.2.1. Rheological Properties of Rubber Mixtures 

**Figure 6:**
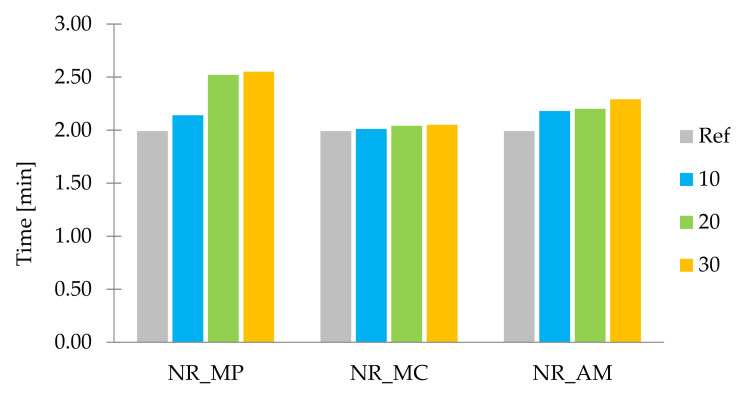
Optimal vulcanization time t_90_ (min).

**Figure 7:**
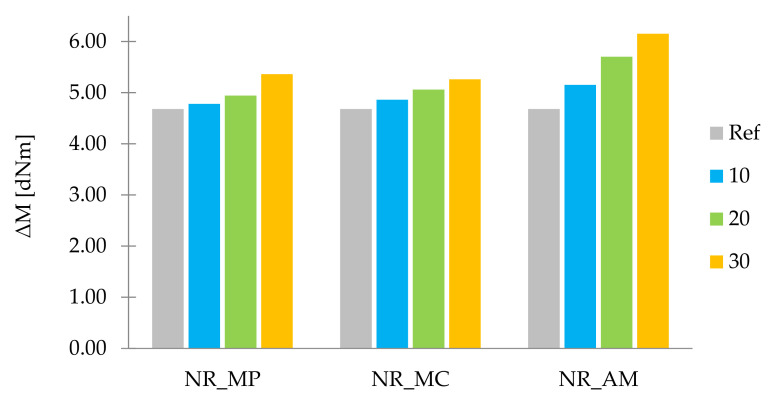
Increase in torque ΔM (dNm).

Non-vulcanized rubber mixtures were subjected to the rheometric tests in order to describe the rheological properties of obtained composites according to the values of two parameters: t_90_ and ΔM. Optimal vulcanization time (t_90_) is a parameter corresponding to the time required for the mixture to reach 90% of maximum torque [97]. An increase in torque (ΔM), as the difference between the maximum (M_max_) and minimum torque (M_min_) [98], was measured to indirectly determine the cross-linking density of samples. The results of tested parameters are presented in the form of a bar graph in Figure 6 and Figure 7.

The linear increase in optimal vulcanization time was recorded for all samples of filled composites compared to the reference sample. The highest values of t_90_ were noticed in the case of peppermint. It may be a result of the absorption of cross-linking system compounds, which reduced the effectiveness of the vulcanization process. Moreover, this could occur due to the low dispersion level of components in the polymer matrix. An extremely slight change of t_90_ was recorded in samples filled with *Matricaria chamomilla* L. This could be related to the high hydrophilicity, which might have caused a low adhesion between filler particles and rubber. Samples containing *Achillea millefolium* L. showed intermediate values of optimal vulcanization time.

By analogy, the increasing tendency of ΔM was performed in the case of filled composites with the increase of filler content in the sample. This tendency might have occurred as a result of the hydrodynamic effect in polymer matrix due to the addition of non-deforming fillers phase. Moreover, increased values of ΔM parameter may be caused by the increase of cross-linking density of composites. These two effects could result in the improvement of mechanical properties.

#### 2.2.2. FTIR Analysis of Composites 

**Figure 8:**
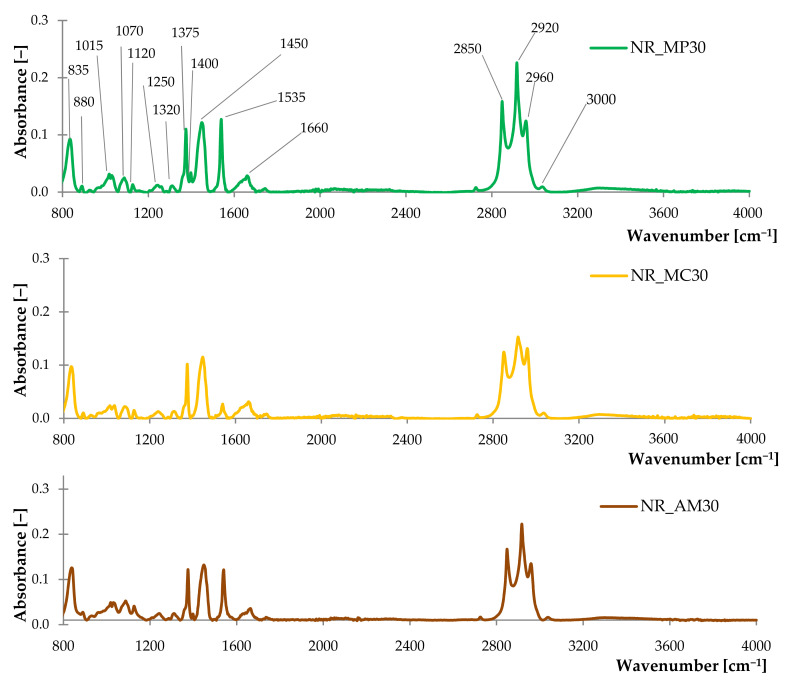
The FTIR spectra of composites filled with 30 phr of peppermint (NR_MP30), German chamomile (NR_MC30) and yarrow (NR_AM30).

The obtained FTIR spectra of the tested composites (Figure 8) indicated the presence of similar functional groups, as in the case of biofillers. Peaks at 2850 cm^−1^, 2920 cm^−1^ and 2960 cm^−1^ were assigned to C−H stretching vibrations. Deformation vibrations of −C=C−H were identified according to the band at 835 cm^−1^ [99]. These groups were characteristic of the presence of natural rubber [100]. An additional group at 1535 cm^−1^ was recorded in the case of composites filled with peppermint and yarrow. It corresponded to the N−H bond probably related to the mercaptobenzothiazole (MBT) [101]. It can be assumed that, in the case of NR_MP and NR_AM, the cross-linking process initiated by active groups from MBT occurred regularly. On the other hand, according to the previous results, German chamomile is a highly hydrophilic plant with increased content of terpenes, terpenoids and phenolic compounds compared to the rest of the fillers. These substances might have caused the interaction with the MBT compound and decreased the registered peak at 1535 cm^−1^.

#### 2.2.3. Thermal Stability of Biocomposites (TGA)

**Figure 9:**
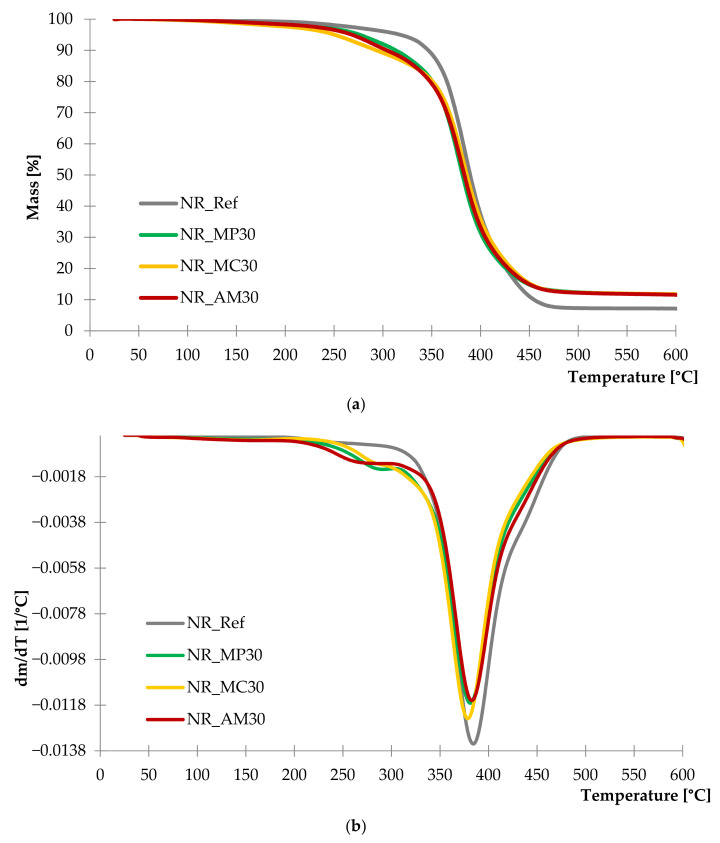
TG (**a**) and DTG (**b**) curves of composites filled with 30 phr of peppermint (NR_MP30), German chamomile (NR_MC30) and yarrow (NR_AM30).

Thermogravimetric analysis was conducted to establish thermal stability of reinforced composites compared to the unfilled system. Characteristic parameters evaluated from the study are presented in Table 3. TG and DTG curves are shown in Figure 9.

According to the DTG and TG curves, the addition of bio-fillers to composites caused a slight decrease in thermal stability compared to the reference sample. The first mass loss related to the thermal decomposition of organic compounds from natural fillers occurred in the case of filled vulcanizates in the range of 230 to 330 °C. Intensive weight loss with the maximum at 380 °C was recorded in all samples and it is assigned to the thermal degradation of natural rubber. Composite filled with *Achillea millefolium* L. showed lower thermal stability at lower temperatures (ca. 210–260 °C) compared to the rest of the samples. On the other hand, it presented the lowest value of Δm_380_. The analysis of the T_10_ parameter showed a rapid mass loss in the case of *Matricaria chamomilla* L. Vulcanizates reinforced with peppermint demonstrated high thermal stability in temperatures below 350 °C. However, it was characterized by the highest value of the mass loss at 380 °C, which indicated the decrease of thermal stability at higher temperatures.

#### 2.2.4. Morphology of Composites (SEM)

**Figure 10:**
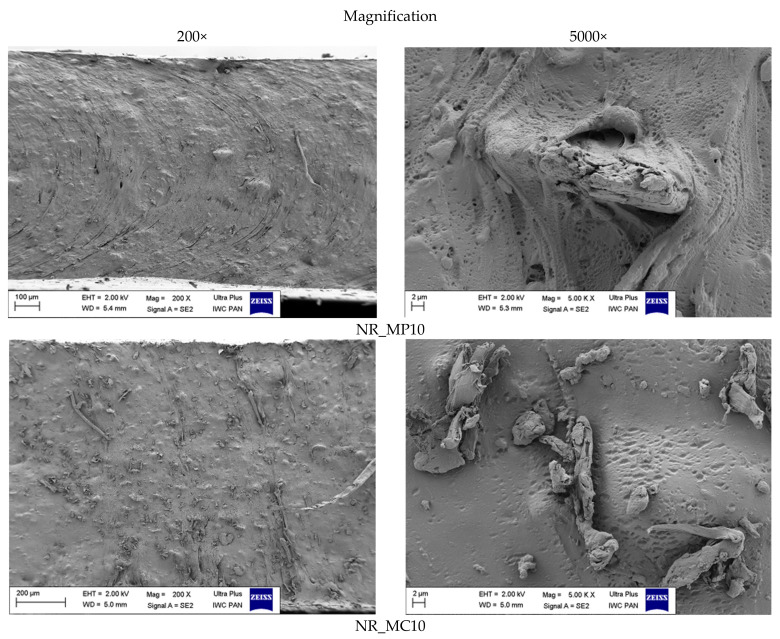

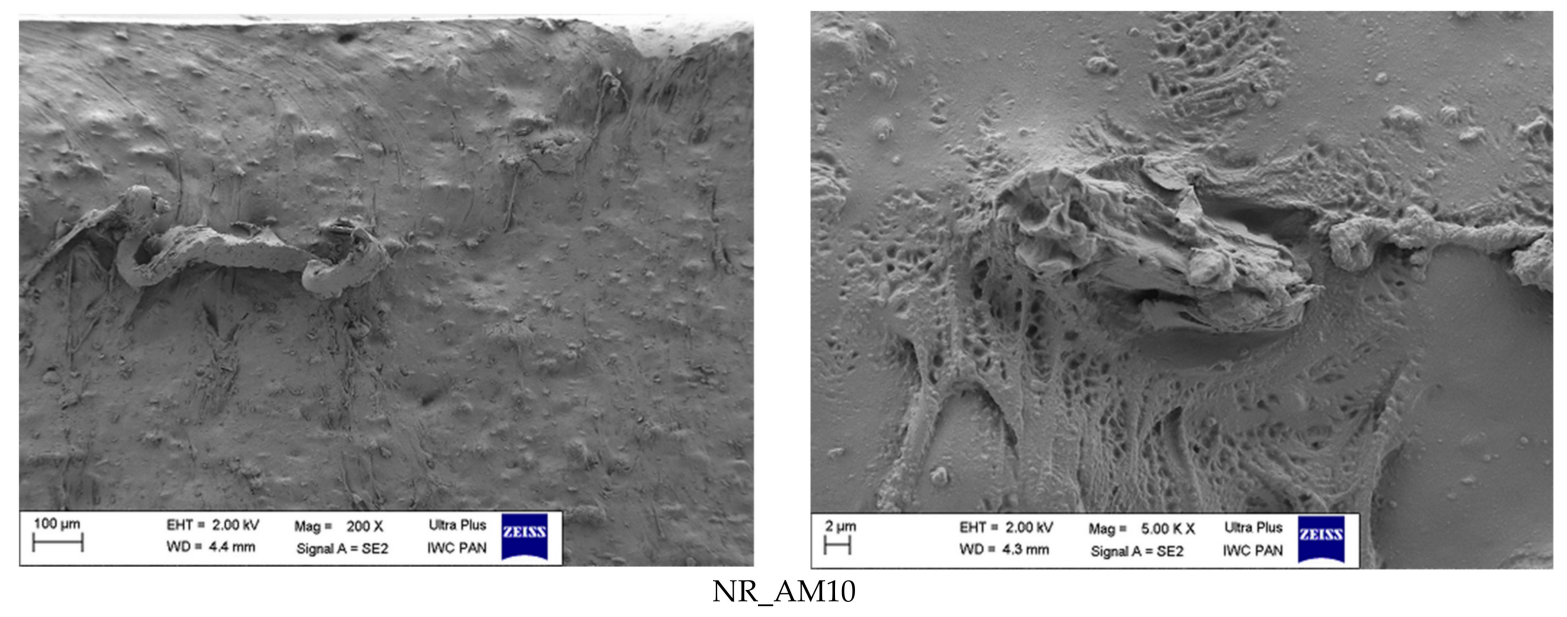
SEM images of biocomposites at the 200× and 5000× magnification.

SEM images were made to present the structure of the obtained biocomposites and the distribution of fillers in the polymer matrix. The results are shown in Figure 10.

The obtained composites differed in the dispersion of the fillers in the material and their adhesion to the rubber. In images of vulcanizates containing peppermint and yarrow, a homogeneous surface was observed, which proved good miscibility of bio-additives in the polymer matrix. Such distribution may strengthen these composites and improve their mechanical properties. Moreover, both fillers showed high adhesion to natural rubber according to the SEM images at the 5.000 magnification. In contrast, some of the *Matricaria chamomilla* L. fibers remained unmixed on the composite surface, creating a heterogeneous blend. The filler particles were mechanically mixed into the matrix, but no adhesion between the two phases was visible. Decreased adhesion might have occurred due to the hydrophilic nature of chamomile, which was determined by the contact angle measurement. These conclusions were correlated with results obtained from rheological studies of biocomposites.

#### 2.2.5. Barrier Properties

**Figure 11:**
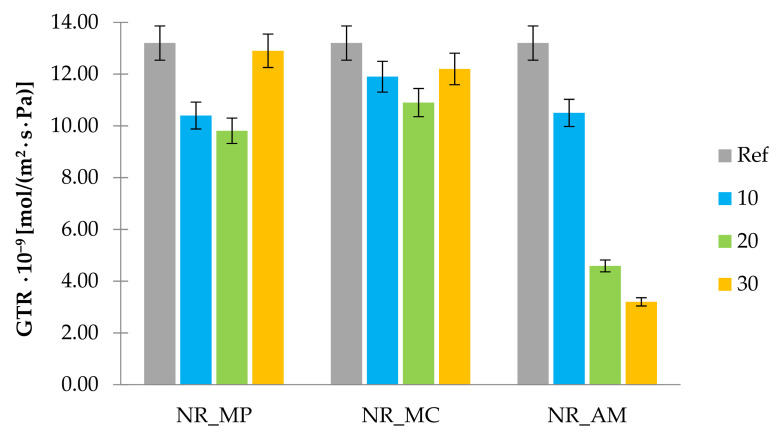
Gas transmission rate of samples.

The gas transmission rate (GTR) was established to measure the air permeability of composite materials and reference sample. Results of obtained GTR are presented in Figure 11.

The analysis of gas transmission rate showed that the addition of peppermint, chamomile and yarrow improved the barrier properties of natural rubber vulcanizates. Biocomposites reinforced with *Mentha piperita* L. and *Matricaria chamomilla* L. showed a similar tendency of the GTR parameter change depending on the bio-additive content in the composite. Peppermint and chamomile added to natural rubber in the increased amount (30 phr) caused deterioration of barrier properties compared to the samples with a lower filling degree. The highest decrease in GTR parameter was measured for samples filled with *Achillea millefolium* L. reaching the minimum value in the case of the highest filling degree. 

Good barrier abilities of NR_AM vulcanizates may occur due to the fibrous structure of filler and its high dispersion in the polymer matrix. Thin and flat yarrow particles may create a so-called “labyrinth effect” for penetrating gas in rubber composite likewise aluminosilicates [102]. Moreover, it can be related to the increased cross-linking density of these composites. 

#### 2.2.6. Cross-Linking Density before and after Aging Processes

To assess the spatial network of natural rubber composites filled with herbs, the cross-linking density was measured. In order to investigate the properties of vulcanizates after the influence of degradation factors, samples were also tested after the simulated ultraviolet (UV) and thermo-oxidative (Therm) aging processes. Obtained values of cross-linking density (ν_e_) are presented in Table 4.

Regardless of the type of filler and its amount in the rubber mixture, the increase in the ν_e_ parameter was noticed compared to the unfilled system. A relatively low cross-linking density was noted for the unaged peppermint vulcanizates in the range of 1.76 to 1.84 mol/cm^3^. Samples filled with *Achillea millefolium* L. demonstrated the most developed spatial structure among all composites, including the impact of aging processes. These results were compatible with previously presented ΔM and GTR parameters, which indicated the noticeable impact of yarrow on biocomposite material properties. The highest ν_e_ value of 3.55 mol/cm^3^ was measured for UV-aged NR_AM vulcanizate with the bio-additive content of 30 phr. 

During aging processes, composites may undergo further cross-linking due to the recombination of free macro-radicals into branched structures [103]. Increased temperature and UV radiation stimulated the occurrence of the described phenomenon. Moreover, the residue of vulcanization agents, under the influence of aging factors, could contribute to a significant increase in cross-linking density. Higher values of ν_e_ after UV aging compared to unaged samples were noticed in the case of peppermint and yarrow composites. On the other hand, natural rubber filled with German chamomile showed greater susceptibility to the influence of increased temperature of thermo-oxidative aging.

#### 2.2.7. Mechanical Properties before and after Aging Processes

**Figure 12:**
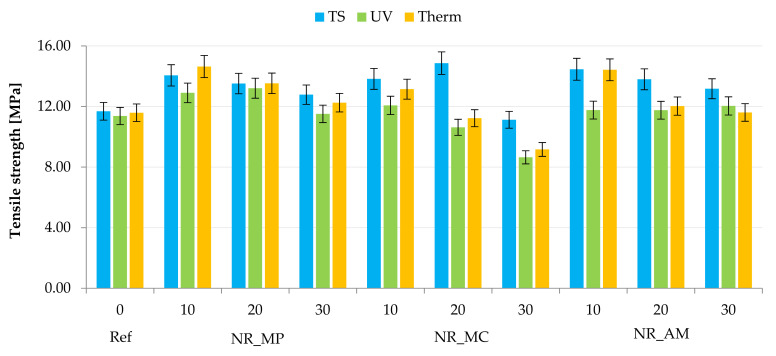
Results of tensile strength measurements of unaged samples (TS), after ultraviolet (UV) and thermo-oxidative (Therm) aging.

**Figure 13:**
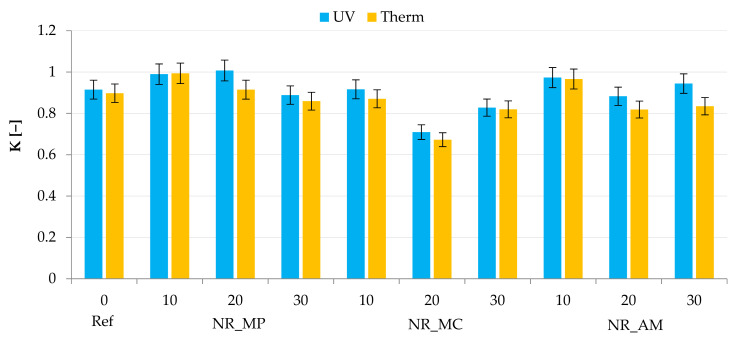
Aging factor K.

Samples were subjected to tensile examinations to provide knowledge about the strength and resistance to stress of biocomposites. The analysis of tensile strength (Figure 12) and elongation at break (Table 5) was conducted for vulcanizates before and after simulated aging processes. Hence, the K coefficient was calculated to present the influence of degradation conditions on the mechanical properties of materials (Figure 13). The obtained results allowed to define the impact of selected bio-additives on the natural rubber composites and predict their lifetime.

In general, the addition of herbs to the natural rubber improved the mechanical properties of unaged composites according to the results of tensile strength and elongation at break. One sample filled with 30 phr of German chamomile presented lower values of TS and Eb compared to the reference sample. The greatest strengthening effect with a tensile strength above 14 MPa was observed for NR_MP10, NR_MC20 and NR_AM10. A linear decrease of TS with the increase of filler content was observed in the case of peppermint and yarrow.

Both aging processes caused a deterioration of mechanical properties, which was related to the degradation of elastomer and as a consequence the increased stiffness and brittleness of materials. The elevated temperature had a slight impact on tensile strength and elongation at break compared to the results for samples after UV radiation. An intensive decrease of TS parameter was recorded for composites filled with 20, 30 phr of German chamomile. These vulcanizates had low resistance to aging processes due to the high hydrophilic properties of the bio-additive. In the case of NR_MP and NR_AM, the elevated temperature or UV radiation caused an additional cross-linking during both aging processes, which did not occur in NR_MC. In most cases, bio-filled vulcanizates demonstrated greater mechanical strength than the reference sample. Therefore, it can be stated that the addition of fillers to natural rubber had a reinforcement effect on biocomposites and may extend polymer lifetime.

These conclusions corresponded to the calculated K factors. The obtained results showed a decrease in mechanical properties of materials after UV and thermo-oxidative aging as the value of the aging coefficient was below the value of 1. Composites filled with *Matricaria chamomilla* L. demonstrated the lowest resistance to the degradation conditions. Moreover, a greater strength effect was observed for vulcanizates with the filler content of 10 phr.

#### 2.2.8. Colour Change

**Figure 14:**
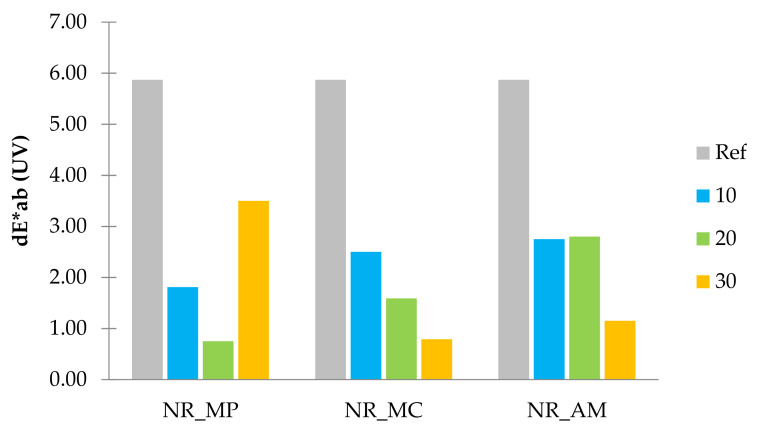
Parameters dE*ab after UV aging.

**Figure 15:**
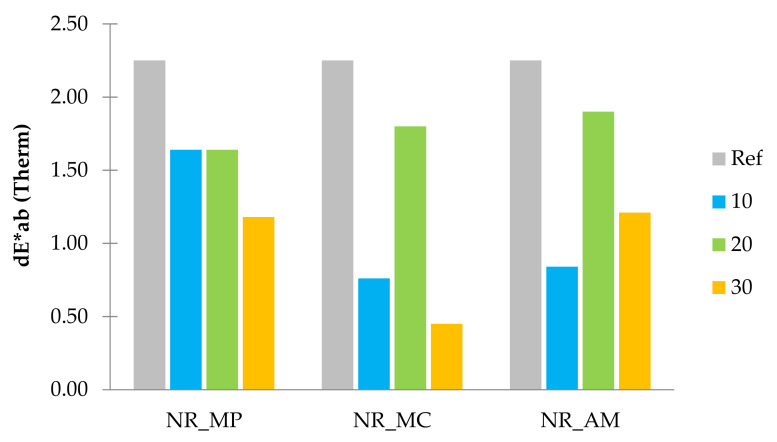
Parameters dE*ab after thermo-oxidative aging.

According to the literature, standard observer notices the difference in colours as the values of dE*_ab_ exceed 3 [104]. The colorimetric measurements were conducted to study the influence of aging factors on biocomposites. Results of obtained colour change (dE*_ab_) after ultraviolet (UV) and thermo-oxidative (Therm) aging are presented in the form of bar graphs in Figure 14 and Figure 15.

Both aging processes had a significant impact on the colour change of elastomeric materials. The addition of peppermint, chamomile and yarrow to the natural rubber resulted in a lower colour change compared to the reference sample. The linear decrease of dE*_ab_ with increasing filler content was noticed in the case of NR_MC and NR_AM after UV radiation and NR_MP after the thermo-oxidative process. The rest of the samples showed irregular colour stability regardless of the plant content. 

It can be stated that all types of the composite were less resistant to UV radiation as the highest values of dE*_ab_ for UV and thermo-oxidative aging were, respectively, 5.90 and 2.25. In the case of the composites, increased resistance to an elevated temperature was provided by the antioxidant nature of used plants and the presence of natural stabilizers in herbs composition.

## 3. Materials and Methods

### 3.1. Materials

Elastomer matrix: natural rubber RSS I (NR) was provided by Torimex Chemicals Sp. z o.o. (Konstantynów Łódzki, Poland). The conventional sulphur curing system consisted of: sulphur (Siarkopol, Tarnobrzeg, Poland), micro-sized zinc oxide (ZnO) (Huta Będzin, Poland), mercaptobenzothiazole (MBT) (Sigma-Aldrich, Poznań, Poland) and stearin (POCH, Gliwice, Poland). The natural fillers were: peppermint (Dary Podlasia Adam Nowicki, Bielsk Podlaski, Poland), German chamomile and yarrow (Ziołowy Zakątek, Grodzisk, Poland).

The raw materials were ground in a 500 mL hardened, stainless steel grinding bowl with twelve 15 mm diameter stainless steel spheres for 40 min at a rotation speed of 300 rpm with a Fritsch Pulverisette 5 planetary mill (Fritsch, Ilztal, Austria).

Dried and powdered plants were screened through a Model 911M PESM laboratory vibratory sieve machine (911 Metallurgy Corp, Langley, BC, Canada). The results of the sieve analysis are shown in Table 6.

### 3.2. Preparation of Rubber Mixtures

Elastomeric mixtures were prepared in three stages. First, natural rubber was plasticized using a Brabender measuring mixer N50 (Brabender Technologie GmBH & Co. KG, Duisburg, Germany) for 4 min with a rotational speed of 40 rpm and a temperature range of 40–60 °C. Then, fillers were dispersed in an elastomer matrix in the same conditions. The last stage of the preparation was introducing a weighed sulfur curing system using a two-roll mill at room temperature. 

Nine biocomposites filled with various content of raw materials and reference sample were prepared for this study. The compositions of elastomeric mixtures are presented in Table 7. 

Constant ratio (%) of constituent materials in *Mentha piperita* L., *Matricaria chamomilla* L., and *Achillea millefolium* L. for each plant by weight including the cross-linking unit, is, respectively: for 10 phr = 8.33%; for 20 phr = 15.39%; for 30 phr = 21.43%.

In order to identify prepared samples, the following abbreviations were used: NR—natural rubber, MP—peppermint, MC—German chamomile, AM—yarrow; numbers: 10, 20, 30—the content of filler: 10 phr, 20 phr, 30 phr.

### 3.3. Methods

UV–Vis spectra of peppermint, chamomile and yarrow were recorded using an Evolution 201/220 UV–Visible spectrophotometer (Thermo Fisher Scientific, Waltham, MA, USA). Measurements of the powdered filler were conducted in the spectral range of 1100 to 200 nm. The test was repeated once for each sample.

Wetting, corresponding to the hydrophilicity or hydrophobicity of natural fillers was defined by the contact angle (Θ) measurement. Powdered substances were formed into tablet-shaped samples with a smooth surface. The measurement was carried out using Dataphysics OCA15EC (DataPhysics Instruments GmbH, Filderstadt, Germany) and was performed by placing a drop of water (1–2 µL) on the tablet. The values of a contact angle correspond to the hydrophilic or hydrophobic nature of the studied material [105]. 

To identify characteristic bonds in studied substances and biocomposites with the filler content of 30 phr, the FTIR spectra were recorded at a resolution of 8 cm^−1^, with 128 scans, over the range of 4000–400 cm^−1^ using the FTIR Nicolet 6700 (Thermo Fisher Scientific, Waltham, MA, USA) reflection ATR technique with an adapter with diamond crystals on a ZnSe plate.

The thermal stability of plant fillers and composites reinforced with 30 phr of peppermint, chamomile and yarrow was studied using the TGA/DSC1 analyzer (Mettler Toledo, Columbus, OH, USA/Greifensee, Switzerland). TGA and DTG curves were recorded in the temperature range of 25 to 600 °C with a 10 °C/min heating rate in a flow of nitrogen at 60 mL/min.

The elastomeric mixtures were subjected to rheometric tests using the MonTech DRPA 300 Rheometer (MonTech Werkstoffprüfmaschinen GmbH, Buchen, Germany). The program was set to subject changes in the torque of the oscillating disc as a function of time at 160 °C. To describe the rheometric properties of studied composites, two parameters were taken from a vulcanization curve: optimal curing time t_90_ (min), which is the time required for the torque to reach 90% and increase in torque ΔM (dNm) as the difference between the maximum (M_max_) and minimum torque (M_min_) during measurement.

Rubber mixtures were cured in steel vulcanization molds of an electrically heated hydraulic press at 160 °C temperature and at 15 MPa pressure for curing time determined from rheometric measurements. 

Biocomposites were subjected to simulated aging processes. Thermo-oxidative simulation was carried out in a forced air dryer Binder Model FED 56 (BINDER GmbH, Tuttlingen, Germany) at 70 °C for 14 days. Ultraviolet degradation was conducted in the UV chamber of Atlas UV 2000 (ATLAS Material Testing Technology GmbH, Duisburg, Germany) in the following aging conditions: the day and night segment: 0.78 W/m^2^; temperature: 60 °C; duration: 72 h. To estimate the resistance of studied vulcanizates to aging processes, their mechanical properties and cross-linking density were determined.

Composites spatial network determined by the cross-linking density was studied according to the solvent-swelling measurements in toluene from the Flory–Rehner Equation (1) [106]:

(1) $$\gamma_{e}=\frac{\ln\left(1-V_{r}\right){+V}_{r}{+\mu V}_{E}^{2}}{V_{0}\left(V_{r}^{\frac{1}{3}}-\frac{V_{r}}{2}\right)}\;$$

where: $\gamma_{e}$—the cross-linking density (mol/cm^3^), V_0_—the molecular volume of solvent (106.7 cm^3^/mol), μ—the Huggins parameter of the NR-solvent interaction calculated from Equation (2):

(2) $${\mu =\mu }_{0}+\beta \cdot V_{r},$$

where: μ_0_—the parameter connected with non-cross-linked/solvent, β—the constant consideration of the impact of cross-linking on parameter polymer/solvent, natural rubber–toluene interaction factor μ_0_ and β were experimentally (μ_0_ = 0.478, β = 0.228); V_r_—the volume fraction of elastomer in the swollen Equation (3):

(3) $$V_{r}=\frac{1}{{1+Q}_{w}\frac{\rho_{k}}{\rho_{r}}},$$

where: Q_w_—weight of equilibrium swelling, Q_k_—density of rubber (g/cm^3^) (0.99 g/cm^3^), Q_r_—density of solvent (g/cm^3^) (0.86 g/cm^3^).

To determine the barrier properties of studied materials, vulcanizates were examined using manometric method according to the ASTM standard D1434. On the basis of through-plane air permeability, tests were conducted in atmospheric air at room temperature. The air permeability was determined by gas transmission rate (GTR) from the following Equation (4) [107]:

(4) $$\mathrm{GTR}=\frac{V_{c}}{R\cdot T\cdot P_{u}\cdot A}\cdot \frac{\mathrm{dp}}{\mathrm{dt}},$$

where: V_c_—volume of low-pressure chamber (L), T—temperature (K), P_u_—the gas pressure in the high-pressure chamber (Pa), A—area permeation of gas through the sample (m^2^), dp/dt—pressure changes per unit time (Pa/s), R—gas constant 8.31 × 10^3^ ((L·Pa)/(K·mol)).

Dumbbell-shaped samples of non-aged and aged composites were prepared to conduct the tensile strength measurements on a testing machine Zwick (model 1435, Ulm, Germany) according to ISO-37. Tests were carried out at room temperature with a cross-head speed of 500 mm/min. 

The aging factor (K) was calculated as a change in the mechanical properties after UV and thermo-oxidative aging processes according to Equation (5) [108]:

(5) $$K\;=\;\frac{{(\mathrm{TS}\cdot E_{b})}_{\mathrm{after}\;\mathrm{aging}}}{{(\mathrm{TS}\cdot E_{b})}_{\mathrm{before}\;\mathrm{aging}}},$$

where E_b_—elongation at break, TS—tensile strength.

Peppermint, German chamomile, yarrow and cryo-fractured samples of composites reinforced with 10 phr of each filler were examined by SEM to estimate the morphology of the materials. Examinations were carried out using the Hitachi TM-1000 (Hitachi Ltd., Tokyo, Japan). 

After the simulated aging processes, elastomer composites were tested according to the PN-EN ISO 105-J01 standard using the Konica Minolta CM-3600d spectrophotometer (Sony, Tokyo, Japan) to measure the colour change of aged samples in comparison with non-aged vulcanizates. The change of colour was calculated according to the CIE-Lab colour space from Equation (6) [109]:

(6) $${\mathrm{dE}}_{\mathrm{ab}\;}^{\;*}=\;\sqrt{\Delta a^{2}+\Delta b^{2}+\Delta L^{2}},\;$$

where: Δa—deviation from the colour of the reference sample in the axis of red–green; Δb—deviation from the colour of the reference sample in the axis of yellow–blue, ΔL—deviation in brightness parameter from the colour of the reference sample.

## 4. Conclusions

In this article, *Mentha piperita* L., *Matricaria chamomilla* L. and *Achillea millefolium* L. were subjected to several different studies to determine the composition, morphology and properties of plants and their possible activity as a bio-fillers in natural rubber composites. The FTIR spectra confirmed the presence of terpenes and terpenoids in the studied herbs. Moreover, the analysis showed that the main building materials were lignocellulosic components. This was also confirmed by the results of thermogravimetric studies and UV–Vis spectra. Moreover, phenolic compounds and chlorophyll were recorded by ultraviolet-visible spectroscopy. The polar and hydrophilic nature of fillers was determined by the goniometric measurements. Diversity in the contact angle of peppermint, German chamomile and yarrow was caused by the differences in morphology visible on SEM images and may be a result of the various components of natural fillers. 

Bio-additives contributed to the extension of the optimal vulcanization time of rubber mixtures during vulcanization, according to the rheometric studies. The highest increase of t_90_ was noticed in the case of peppermint in contrast to the samples filled with German chamomile. In addition, the increasing tendency of ΔM parameter was determined. The analysis of FTIR spectra of composites indicated the presence of natural rubber and components characteristic for added plants. According to the TG and DTG curves, a slight decrease of thermal stability was established for filled vulcanizates compared to the reference sample. The best adhesion to natural rubber occurred in the case of peppermint and yarrow. The addition of bio-fillers improved barrier properties compared to the unfilled system, especially for vulcanizates filled with *Achillea millefolium* L. The increase of cross-linking density was observed in all reinforced composites, including the results after simulated aging. Conducted studies of tensile strength and elongation at break proved the reinforcement effect of natural fillers in both unaged and aged biocomposites compared to the reference sample. A linear increase of tensile strength with the decreased filler content was observed for vulcanizates filled with peppermint and yarrow. The lowest values of aging coefficient K were calculated for composites with the addition of *Matricaria chamomilla* L. and an unfilled system. The colour change test results indicated the higher resistance to elevated temperature rather than ultraviolet radiation due to the antioxidant nature of bio-additives.

The addition of *Achillea millefolium* L. ensured the highest cross-linking density, and thus decreased the gas transmission rate and improved the mechanical properties of the composites. Among the tested plants, yarrow had the most advantageous influence on elastomer mixture properties. Particles of *Mentha piperita* L. added to the natural rubber with cross-linking system created a homogeneous blend, which provided an increase of tensile strength and improved the barrier and mechanical properties compared to the reference sample. On the other hand, the cross-linking density achieved the lowest values among the rest of composites. Composites filled with *Matricaria chamomilla* L. showed increased cross-linking density and tensile strength. The gas permeability decreased compared to the reference sample; however, the values of GTR were higher than the rest of reinforced biocomposites. All tested vulcanizates filled with bio-additive presented an extension of optimal vulcanization time and decreased thermal stability over 200 °C compared to the reference sample. The presented study constitutes a scientific novelty in the field of composite materials, as there are currently no literature reports on the characterization of *Mentha piperita* L., *Matricaria chamomilla* L. and *Achillea millefolium* L. as a natural filler for elastomer biocomposites. Physico-chemical characteristics proved the reinforcement effect of selected plants on natural rubber-based composites. According to the results, peppermint, German chamomile and yarrow may find its application in polymer technology as active or semi-active fillers.

## Figures and Tables

**Table 1:** Characteristic functional groups registered by FTIR analysis of fillers [84].

Peak Assignments and Type of Vibration	Wavenumber [cm^−1^]
*v* (O–H) phenols and alcohols, –C=O*_w_* (overtone) and *v* (=C–H*_vw_*)	3650–3200
*v* (C–H) vinyl & acrylic	3100–3010
*v* (C–H) aliphatic and ν*_as_*(–C–H*_m_*, –CH_3_, –CH_2_)	2970–2800
*v* (C=O)	1740
*v* (C=O)	1730–1690
*v* (–C=O*_vw_*) in acids	1708
*v* (C=C) alkenes, amide	1680–1610
*v_vw_*(–C=C–, cis-) and δ(–OH)	1675–1648
*v* (COOH) *v* (C=C) stretching	1634–1643 1600–1620
*v*(C=C) aryl, *d_vw_*(–CH_2_) and (–CH_3_) bending (scissoring) or *v_vw_*(–C–H) bending (rocking)	1600–1500
*v*(C–C) aliphatic	1500–600
*sv*(C=C) aromatic	1441
*d*(C–H) aliphatic	1370–1340
*v_w_*_,*m*,*vw*_(–C–H, –CH_3_)	1372/1337
*v_as_*_,*s*_(C–O, C–C, –C–O–C–)	1285–1150
*v*(C–O), (C–C)	1020–1030
*v_m_*(–C–O) or *d_m_*(–CH_2_–)	1285/1244
*v_st_*(–C–O) or *d_st_*(–CH_2_–)	1116
ν*_m_*(–C–O)	1094
*d_a_*(C–O–C)	1060
*v_m_*_,*vw*_(–C–O)	1044/1023
*d_w_*(–HC=CH–)	991/923
*v*(C=C)	880

Abbreviations: *v*—stretching vibrations; *d*—deformation vibrations; *s*—symmetric; *as*—asymmetric; *st*—strong; *vst*—very strong, *w*—weak; *sv*—skeletal vibration; *a*—axial.

**Table 2:** Thermal stability of fillers determined by TGA.

Sample	T_10_ ^1^ (°C)	T_50_ ^2^ (°C)	Δm_100_ ^3^ (%)	Δm_320_ ^4^ (%)	Residue at 600 °C ^5^ (%)
MP	203.33	343.75	4.99	41.63	31.30
MC	223.33	328.34	3.83	44.70	28.53
AM	192.50	322.50	3.18	48.67	27.93

^1^ T_10_—temperature of 10% mass loss, ^2^ T_50_—temperature of 50% mass loss, ^3^ Δm100—mass loss at 100 °C of the sample, ^4^ Δm320—mass loss at 320 °C, ^5^ Residue at 600 °C—mass of the sample at 600 °C.

**Table 3:** Thermal stability of composites determined by thermogravimetric analysis.

Sample Name	T_10_ ^1^ (°C)	Δm_380_ ^2^ (%)	Residue at 600 °C ^3^ (%)
Ref. Sample (NR)	362.64	41.02	7.13
NR_MP	328.71	51.82	11.57
NR_MC	307.78	46.38	11.74
NR_AM	318.25	54.21	11.56

^1^ T_10_—temperature of 10% mass loss, ^2^ Δm_380_—mass loss at 380 °C, ^3^ Residue at 600 °C—mass of the sample at 600 °C.

**Table 4:** Results of cross-linking density.

Sample	Filler Content (phr)	ν_e_ × 10^5^ (mol/cm^3^)		
		Ref	UV	Therm
Ref. sample (NR)	0	1.71 ± 0.01	1.90 ± 0.01	1.81 ± 0.02
NR_MP	10	1.76 ± 0.01	2.12 ± 0.02	2.03 ± 0.02
	20	1.82 ± 0.03	2.29 ± 0.04	2.07 ± 0.02
	30	1.84 ± 0.03	2.63 ± 0.03	2.50 ± 0.04
NR_MC	10	1.84 ± 0.02	2.19 ± 0.03	2.40 ± 0.05
	20	1.94 ± 0.03	1.94 ± 0.05	2.21 ± 0.04
	30	2.01 ± 0.04	2.09 ± 0.04	2.26 ± 0.03
NR_AM	10	2.07 ± 0.01	2.34 ± 0.02	2.43 ± 0.03
	20	2.09 ± 0.03	3.21 ± 0.04	2.46 ± 0.03
	30	2.36 ± 0.03	3.55 ± 0.03	2.56 ± 0.04

**Table 5:** Elongation at brake before (Eb) and after ultraviolet (Eb_UV_) and thermo-oxidative (Eb_Therm_) aging.

Sample	Filler Content (phr)	Eb (%)	Eb_UV_ (%)	Eb_Therm_ (%)
Ref. Sample (NR)	0	656.04 ± 3.48	736.90 ± 9.56	593.53 ± 6.46
NR_MP	10	740.99 ± 2.12	798.99 ± 3.49	707.03 ± 5.25
	20	752.50 ± 2.21	775.71 ± 6.13	687.38 ± 5.68
	30	754.34 ± 3.25	743.81 ± 1.59	675.81 ± 5.91
NR_MC	10	716.54 ± 4.94	751.65 ± 9.45	655.90 ± 7.34
	20	735.85 ± 6.71	730.04 ± 4.44	655.14 ± 10.29
	30	640.91 ± 9.11	682.60 ±11.07	637.35 ±12.34
NR_AM	10	671.99 ± 1.49	739.40 ± 4.47	598.76 ± 1.52
	20	712.27 ± 3.33	737.79 ± 3.05	668.72 ± 0.38
	30	683.16 ± 2.54	705.77 ± 7.61	647.15 ± 2.34

**Table 6:** Sieve analysis of fillers.

Fraction [mm]	Sieve Sizes [mm]	MP ^1^		MC ^2^		AM ^3^	
		(g)	(%)	(g)	(%)	(g)	(%)
1.000–2.000	1.000	1.30	2.60	0.29	0.57	0.71	1.41
0.500–1.000	0.500	9.54	19.07	10.57	21.13	11.68	23.35
0.250–0.500	0.250	28.32	56.64	30.06	60.12	29.22	58.43
0.125–0.250	0.125	10.11	20.21	8.69	17.38	7.81	15.61
0.065–0.125	0.065	0.74	1.48	0.40	0.80	0.60	1.20
	Σ	50.00	100.00	50.00	100.00	50.00	100.00

^1^*Mentha piperita* L., peppermint; ^2^ *Matricaria chamomilla* L., chamomile; ^3^ *Achillea millefolium* L., yarrow.

**Table 7:** The composition of elastomeric mixtures.

Sample Name	Filler	NR	Stearin	ZnO	MBT	Sulfur
	(phr ^1^)					
Reference Sample (NR)	0	100	1	5	2	2
NR_MP10	10	100	1	5	2	2
NR_MP20	20	100	1	5	2	2
NR_MP30	30	100	1	5	2	2
NR_MC10	10	100	1	5	2	2
NR_MC20	20	100	1	5	2	2
NR_MC30	30	100	1	5	2	2
NR_AM10	10	100	1	5	2	2
NR_AM20	20	100	1	5	2	2
NR_AM30	30	100	1	5	2	2

^1^ phr—parts per hundred parts of rubber.

## Data Availability

Data sharing not applicable.
